# Supplementary material for: Sequential regulatory activity prediction across chromosomes with convolutional neural networks
Source: Genome Res. 2018 May;28(5):739–50. doi: 10.1101/gr.227819.117 (PMC5932613; doi:10.1101/gr.227819.117)
Supplement: Supplemental Material [file supp_28_5_739__index.html]

Sequential regulatory activity prediction across chromosomes with convolutional neural networks — Supplemental Material 

# Sequential regulatory activity prediction across chromosomes with convolutional neural networks

## Supplemental Material

- Supplemental\_Table\_S1.xls
- Supplemental\_Material.pdf
- Supplemental\_Source\_Code.zip
